# The Optimisation of Pseudotyped Viruses for the Characterisation of Immune Responses to Equine Influenza Virus

**DOI:** 10.3390/pathogens5040068

**Published:** 2016-12-15

**Authors:** Simon D. Scott, Rebecca Kinsley, Nigel Temperton, Janet M. Daly

**Affiliations:** 1 Viral Pseudotype Unit, School of Pharmacy, University of Kent, Central Avenue, Chatham Maritime ME4 4TB, UK; S.D.Scott@kent.ac.uk (S.D.S.); rk320@kent.ac.uk (R.K.); n.temperton@kent.ac.uk (N.T.); 2 School of Veterinary Medicine and Science, University of Nottingham, Sutton Bonington LE12 5RD, UK

**Keywords:** equine influenza, pseudotyped virus, neutralisation assay

## Abstract

Pseudotyped viruses (PVs) produced by co-transfecting cells with plasmids expressing lentiviral core proteins and viral envelope proteins are potentially powerful tools for studying various aspects of equine influenza virus (EIV) biology. The aim of this study was to optimise production of equine influenza PVs. Co-transfection of the HAT protease to activate the haemagglutinin (HA) yielded a higher titre PV than TMPRSS2 with the HA from A/equine/Richmond/1/2007 (H3N8), whereas for A/equine/Newmarket/79 (H3N8), both proteases resulted in equivalent titres. TMPRSS4 was ineffective with the HA of either strain. There was also an inverse relationship between the amount of protease-expression plasmids and the PV titre obtained.  Interestingly, the PV titre obtained by co-transfection of a plasmid encoding the cognate N8 NA was not as high as that generated by the addition of exogenous neuraminidase (NA) from *Clostridium perfringens* to allow the release of nascent PV particles. Finally, initial characterisation of the reliability of PV neutralisation tests (PVNTs) demonstrated good intra-laboratory repeatability. In conclusion, we have demonstrated that equine influenza PV production can be readily optimised to provide a flexible tool for studying EIV.

## 1. Introduction

Pseudotyped viruses (PVs) are increasingly being used to study a wide range of aspects of virus biology such as receptor binding, antiviral screening, vaccine evaluation, and seroepidemiology (reviewed in [1]). A typical approach for generating PVs is to co-transfect ‘producer’ cells with plasmids expressing (i) the envelope protein of a virus of interest; (ii) proteins of a ‘core’ virus, often a retrovirus; and (iii) a reporter transgene. The envelope protein enables the virus to enter ‘target’ cells permissive to the virus of interest. During cell transduction, the reporter transgene becomes integrated into the genome of the cell (e.g., via lentivirus vector components) and is expressed, permitting quantification. Influenza A viruses express two surface glycoproteins; haemagglutinin (HA) and neuraminidase (NA). The HA mediates binding to receptors on host cells prior to internalisation of the virus and is a major target of virus neutralising antibodies. In order for influenza virus particles to become infectious, the newly synthesised HA polyprotein (HA0) has to undergo precise protease-mediated cleavage into two subunits (HA1 and HA2). This event exposes a ’fusion peptide’ crucial for endosomal membrane fusion (reviewed in [2]). In nature, this virion activation is performed by intracellular enzymes. For most influenza A subtypes, including the equine H3N8 subtype, this cleavage relies on trypsin or trypsin-like proteases such as Transmembrane Protease Serine S1 member (TMPRSS2 or epitheliasin) or Human Airway Trypsin-like protease (HAT or TMPRSS11D) [3]. Therefore, to generate infective influenza PV particles, co-transfection of a fourth plasmid expressing protease is required (reviewed in [1]).

Accumulation of amino acid changes in the HA by a process known as antigenic drift means that antibodies raised by exposure to an earlier strain (either through infection or vaccination) may no longer be fully protective against a newer strain. Thus, both human and equine influenza virus (EIV) vaccine strains are updated regularly, albeit more frequently for human influenza. Analysis of the serological response to vaccination with updated vaccines is an important component of the process for updating vaccines. Although neutralisation tests are generally regarded as the gold standard for measuring antibody titres, the haemagglutination inhibition (HI) test remains the mainstay of human influenza vaccine evaluation. However, the single radial haemolysis (SRH) assay is more widely used to evaluate equine influenza vaccines as SRH levels correlate with protective efficacy of vaccines (reviewed in [4]). We previously demonstrated a 65% correlation between the results of a neutralisation test using an EIV PV and an SRH assay, with the pseudotyped virus neutralisation test (PVNT) appearing slightly more sensitive [5]. However, the PV used only expressed the EIV HA, and a recent study highlighted the importance of the antibody response to NA, measured by a neuraminidase inhibition test, in determining protection against infection for human influenza [6].

The NA protein is essential for release of nascent virus particles; therefore, in order to harvest HA-pseudotyped virus particles from cell culture media during production, it is necessary to provide NA. Typically, exogenous NA, derived from commercially purified sources (e.g., *Clostridium perfringens*) is added to the cell culture medium after plasmid-transfection. However, influenza PVs can be generated by incorporating NA-expressing plasmids in the co-transfection procedure, thereby producing particles with both HA and NA on their surface [7,8,9,10,11,12].

In this study, we sought to optimise production of PVs by first employing different proteases with the particular aim of maximizing titre to enable large-scale studies to be performed using a single PV batch. We also generated a PV co-expressing the EIV H3 HA and N8 NA from the same strain and compared the titre obtained with that obtained by providing exogenous NA. Finally, we performed some initial characterisation of intra-laboratory repeatability of the PVNT.

## 2. Results

### 2.1. The Effect of Different Proteases on Equine Influenza H3 PV Titre

**Figure 1:**
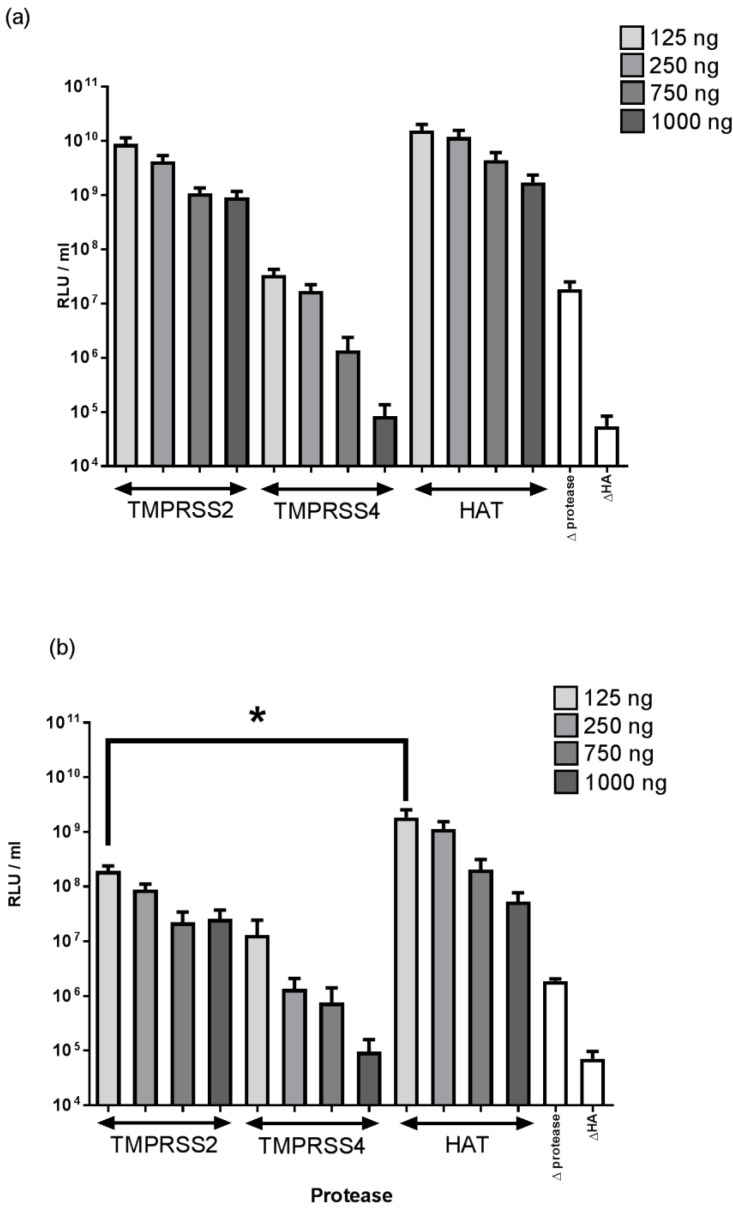
Titres obtained after transduction of 293T target cells with pseudotyped viruses produced via co-transfection of three different protease-expressing plasmid vectors (TMPRSS2, TMPRSS4, HAT) using a range of protease plasmid masses for two strains: (**a**) A/equine/Newmarket/1979 (H3N8); (**b**) A/equine/Richmond/1/2007 (H3N8). Controls had no protease-expressing plasmid (Δ protease) or no HA plasmid (ΔHA) added during transfection. Titres are expressed as mean relative luminescence units (RLU) per mL with error bars indicating the standard error of the mean. Statistically significant differences were calculated using an unpaired t-test and indicated by * (*p* < 0.05).

Figure 1 shows the relative titres of PVs generated for two strains of equine influenza (A/equine/Newmarket/1979 and A/equine/Richmond/1/2007) using different quantities of TMPRSS2, TMPRSS4, and HAT (TMPRSS11D) protease plasmids. In all cases, the highest titre was achieved using the lowest amount of protease plasmid (125 ng per transfection). There was no significant difference in titre obtained using 125 ng of the TMPRSS4 plasmid and the negative control in which no protease was provided (*p* > 0.05), suggesting that TMPRSS4 does not cleave the equine H3 HA. In contrast, 125 ng of the TMPRSS2 and HAT plasmids resulted in high PV titres (>1 × 10^8^ Relative Luminescence Units/RLU) for both H3 strains. For Richmond/2007, HAT yielded a significantly higher titre PV than TMPRSS2 (*p* = 0.042). However, there was no significant difference in PV titre using HAT or TMPRSS2 for the Newmarket/79 strain (*p* = 0.217).

### 2.2. Influence of Source of Neuraminidase on PV Titre

**Figure 2:**
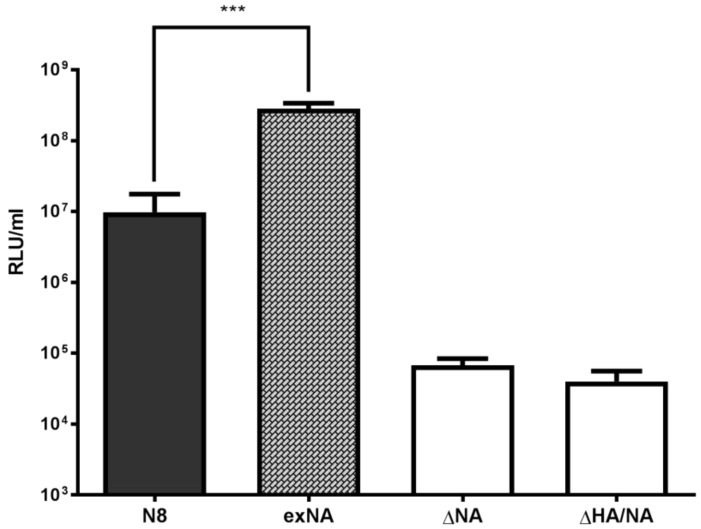
Titres of influenza A/equine/Richmond/1/2007 (H3N8) pseudotyped virus generated by co-transfection with plasmids expressing HA and N8 or by addition of an exogenous source of NA (exNA) 24 h post-transfection. Negative controls had H3 HA but no NA added (ΔNA) or neither NA or HA added (ΔHA/NA). Titres are expressed in relative luminescence units (RLU) per mL. Error bars show standard error of the mean of 8 replicates. The statistically significant difference (Mann–Whitney test) between mean RLU/mL of N8 plasmid and the addition of exNA is indicated by *** (*p* < 0.001).

The titre of PVs obtained using a standard production protocol in which exogenous (*Clostridium perfringens*) NA is added was compared with the titre obtained with the incorporation of influenza NA proteins into PV particles (Figure 2). The titre was similar (around 10^5^ RLU/mL) for negative controls with no NA provided (ΔNA) or no surface protein provided (ΔNA/HA). Addition of exogenous NA resulted in a significantly higher titre PV than the negative controls (*p* = 0.001), demonstrating that NA is essential for release of PV particles from producer cells. Providing NA of the N8 subtype by co-transfecting plasmid resulted in a high titre PV. Interestingly, the PV with N8 NA from the same virus strain as the HA was significantly higher than the Delta NA control, but was lower in titre than the PV produced with exogenous NA.

### 2.3. Repeatability of Pseudotyped Virus Neutralisation Tests (PVNTs)

**Figure 3:**
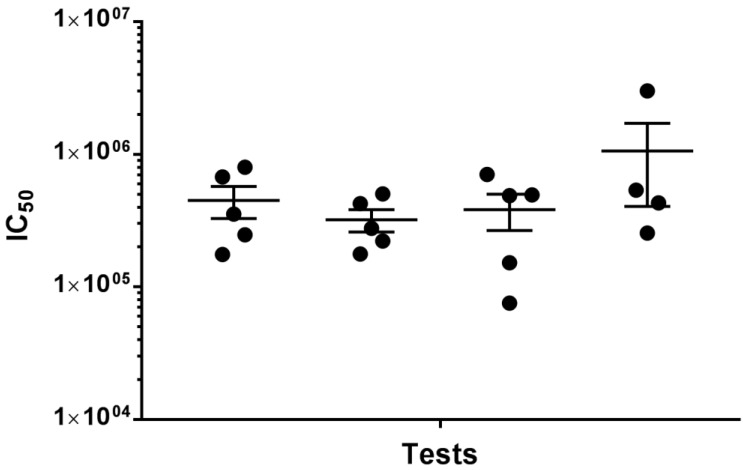
Neutralisation titres (50% inhibitory concentration, IC_50_) obtained using a positive control serum in four independent PVNTs using a PV expressing A/equine/Richmond/1/2007 (H3N8) HA (produced with an endogenous HAT protease-encoding plasmid and an exogenous source of NA). The PV RLU input ranged from 1.83 to 3.43 × 10^5^, and the serum dilution range was 1:800 to 1:6,553,600. Individual data points represent the IC_50_ calculated from each of the 5 (or 4) internal PVNT repeats. Horizontal line indicates mean IC_50_ and whiskers represent the standard error of the mean.

Using a positive control serum sample and a PV expressing the HA of A/equine/Richmond/07 (H3N8), PVNTs were performed on four independent occasions (Figure 3). One-way ANOVA of the antibody titres expressed as IC_50_ (the reciprocal of serum dilution required for 50% PV neutralisation) revealed no significant differences between the repeats (*p* = 0.318).

## 3. Discussion

Pseudotyped virus neutralisation tests are being increasingly used to measure antibody responses to influenza A viruses for experimental studies. However, if they are to be more widely adopted, including outside the research laboratory, PVNTs will have to demonstrate at least equivalent utility to established assays. As variability can occur when the reagents used change from assay to assay, we investigated whether different proteases to cleave, and thus activate the HA, could be used to optimise the titre of PV produced for larger or high-throughput serological studies. This would reduce the necessity to use different batches of PV within a single study. Our results indicated that human HAT and TMPRSS2 proteases can efficiently cleave both equine influenza H3 strain HAs tested. Higher titres of the Richmond/07 PV were obtained using HAT than with TMPRSS2 while titres obtained with Newmarket/79 were equivalent using HAT or TMPRSS2. The use of TMPRSS2 to successfully generate PVs expressing HA from various subtypes has been previously reported [13,14], and TMPRSS2 was used to generate the first reported equine influenza PV [5]. The HAT protease has also been used previously to generate PVs expressing human H3, H1, and H5 [14]. However, the titre of equine H3 PVs generated using TMPRSS4 was no better than the negative control lacking protease. Chaipan et al. (2009) demonstrated that TMPRSS4 could be used to produce a PV bearing the A/South Carolina/1/1918 (H1N1) pandemic strain HA, using the same expression plasmid used in this study [15]. The HA cleavage sites of the H1 HA from the 1918 strain and both equine H3 HAs used in the current study are monobasic (Q-X-R), specifically QIR in the case of both equine H3 strains. However, it is possible that amino acid changes in the vicinity of the cleavage site may influence the ability of different proteases to recognise and cleave the HA from different virus subtypes or strains.

Although optimisation of the HA and core plasmids is extensively reported (reviewed in [1]), there is an absence of published work describing the impact of varying the amounts of protease used during PV production. An inverse correlation between the amount of protease-expressing plasmid used and the titre of PV obtained was demonstrated here. Thus, it is clear that plasmid titration is essential to maximise the concentration of activated PV particles.

We next investigated the impact on PV titre of employing a different source of NA. In the standard protocol, an exogenous source of NA (commercially purified *Clostridium perfringens* enzyme) is used, but NA can be provided by inclusion of NA-expressing plasmids during co-transfection. Although it would have been more cost-effective to use an NA expression plasmid, use of exogenous NA was more efficient. However, it may be desirable to generate a PV expressing both HA and NA. For example, a comparison of neutralisation titres obtained with a PV expressing HA alone and one expressing both HA and NA would enable the measurement of the contribution of anti-neuraminidase antibodies to a protective immune response. For example, NA-inhibiting antibody titres could be measured by adding serial dilutions of serum to producer cells after transfection and measuring the reduction in titre of released PV in a similar approach to that used to determine neuraminidase inhibitor activity [16].

It is believed that only viruses with balanced enzymatic activity of the NA and HA binding avidity can survive in nature and that the HA and NA undergo compensatory co-evolution during antigenic drift in order to maintain this balance [17,18]. Nonetheless, different combinations of HA and NA may differ in their compatibility. A study using reverse genetics to generate reassortant viruses found that a reassortant with an H9N2 background and avian N3 NA replicated more efficiently in mice than the parental H9N2 virus but less efficiently in chickens, suggesting that an H9N3 reassortant might more readily become established in a mammalian host [19]. The N3 NA also had a significantly higher enzymatic activity than the N2 NA. In another study, supplying endogenous N1, N3, N4, and N9 NA (as co-transfected plasmids), all resulted in high titre PV production with the A/equine/Richmond/1/2007 HA (data not shown) with the N1 NA resulting in a PV titre equivalent to using exogenous NA. These results emphasise that whether it is preferable to provide neuraminidase activity exogenously or endogenously should be assessed on a case-by-case basis, depending on the application for which the PV is required.

Another point to consider is that the sialic acid receptor composition on the human-derived HEK293T/17 cells, used as the target cell line for the PVNT, is likely to be different to that of equine cells. An equine dermal cell line kindly provided by Dr. Pablo Murcia (University of Glasgow) was trialled in a PV titration assay, but the production titre was reduced compared to HEK293T/17 cells (data not shown). Equine cell lines are not easy to obtain commercially; isolation of equine influenza viruses is typically done in embryonated hens’ eggs.

Finally, in a separate study, we tested the repeatability of the PVNT. Several studies have compared PVNT with other serological assays [1], but there is a paucity of similar published studies directly examining robustness of the PVNT. Four independent experiments were conducted on different days using the same batch of an A/equine/Richmond/07 (H3N8) HA-only PV. Results revealed no statistically significant differences in IC_50_ values between the tests, demonstrating intra-laboratory consistency of the assay when performed by the same researcher. However, further studies are required, including an inter-laboratory assessment of the reproducibility of the assay. This testing is necessary if the PVNT is to become more widely adopted for equine influenza scientific work (e.g., studies of the impact of changes in the HA on antigenicity) and clinical applications such as equine vaccine evaluation, using a safe, reliable, flexible, biologically relevant system.

## 4. Materials and Methods

### 4.1. Generation of H3 Pseudotyped Viruses

The full-length HA genes of A/equine/Newmarket/1979 (H3N8) and A/equine/Richmond/2007 (H3N8) were kindly provided by Dr. Adam Rash and Dr. Debra Elton (Animal Health Trust, Newmarket, UK) who PCR-amplified the HA gene using the custom primers (Invitrogen) described in [5]. The PCR fragments were digested with restriction enzymes *EcoRV/BamHI* and *BamHI/XhoI* (Thermo Scientific) for A/equine/Newmarket/1979 and A/equine/Richmond/2007, respectively, and cloned into the pI.18 expression plasmid. Both gene sequences were confirmed by bidirectional custom sequencing (GATC Biotech) using primers binding to the arms of the pI.18 vector.

Influenza pseudotyped lentivirus particles expressing the firefly luciferase reporter gene were generated by plasmid co-transfection as previously described [5] with the following variations. Different protease plasmids were used: pCAGGS-TMPRSS2, pCAGGS-TMPRSS4, and pCAGGS-HAT. The HAT and TMPRSS2 proteases were kindly provided by Dr. Hans Dieter Klenk (Philipps-Universität, Marburg, Germany) and the TMPRSS4 plasmid by Dr. Stefan Pohlmann (Leibniz-Institut für Primatenforschung, Göttingen, Germany). Polyethylenimine (PEI) was used as the transfection reagent. Neuraminidase was supplied either exogenously, by the addition of 1U of soluble *Clostridium perfringens* NA (Sigma) 24 h after transfection, or by transfection of an additional plasmid (pI.18) expressing the relevant viral NA. The NA gene of A/equine/Richmond/1/2007 (H3N8), GenBank accession number KF559336 was synthesised by Genscript and cloned into pUC57 before sub-cloning into pI.18 using restriction enzymes *BamHI*/*XhoI*.

### 4.2. Pseudotyped Virus Neutralisation Test (PVNT)

The PVs were titrated and neutralisation tests performed essentially as described previously using positive control serum from a hyper-immunised experimental pony and negative control serum from two influenza-naïve horses [5].

### 4.3. Statistical Analysis

Data were expressed as mean ± standard error of the mean. Differences between two data sets were tested with an unpaired *t*-test unless normal distributions could not be assumed, in which case the Mann–Whitney test was used. ANOVA was used to test repeated measures. *p*-values < 0.05 were considered statistically significant. PVNA IC_50_ values were calculated by normalising raw RLU values to assay controls and performing a non-linear regression analysis using GraphPad Prism version 7.01 for Windows, GraphPad Software, La Jolla California USA, www.graphpad.com. Raw data are available on request.

## Abbreviations

The following abbreviations are used in this manuscript:

EIV	equine influenza virus
HA	haemagglutinin
HAT	human airway trypsin-like protease
NA	neuraminidase
PV	pseudotyped virus
PVNT	pseudotyped virus neutralisation test
TMPRSS2/4	Transmembrane Protease Serine S1 member 2/4
